# Neovascular glaucoma: a review

**DOI:** 10.1186/s40942-016-0051-x

**Published:** 2016-11-14

**Authors:** Gustavo B. Rodrigues, Ricardo Y. Abe, Camila Zangalli, Savio L. Sodre, Flavia A. Donini, Danilo C. Costa, Andre Leite, Joao P. Felix, Marcelo Torigoe, Alberto Diniz-Filho, Homero Gusmão de Almeida

**Affiliations:** 1 Department of Ophthalmology, Faculdade de Ciências Médicas - UNICAMP, University of Campinas, Caixa Postal - 6111, Campinas, SP 13083-970 Brazil; 2 Department of Ophthalmology and Otorhinolaryngology, Federal University of Minas Gerais, Belo Horizonte, Brazil

**Keywords:** Neovascular glaucoma, Refractory, Anti-VEGF, Diabetes, Central retinal vein occlusion

## Abstract

Neovascular glaucoma (NVG) is a secondary glaucoma generally associated with poor visual prognosis. The development of new vessels over the iris and the iridocorneal angle can obstruct aqueous humor outflow and lead to increased intraocular pressure. The underlying pathogenesis in most cases is posterior segment ischemia, which is most commonly secondary to proliferative diabetic retinopathy or central vein retinal occlusion. The neovascularization process in the eye is driven by the events that alter the homeostatic balance between pro-angiogenic factors, such as the *vascular endothelial growth factor* and anti-angiogenic factors, such as the pigment-epithelium-derived factor. Early diagnosis of this condition through slit lamp examination of the iris, iridocorneal angle and retina can help to avoid the development of goniosynechia and obstruction of aqueous humor outflow, with consequent intraocular pressure elevation. Historically, NVG treatment was focused on reducing the posterior segment ischemic process that caused the formation of new vessels, through panretinal photocoagulation. Recently, several studies have investigated the application of intravitreal anti-VEGF therapies in NVG. If clinical treatment with the use of hypotensive topical drops is not sufficient, laser and/or surgical procedures are required for intraocular pressure control.

## Introduction

Neovascular glaucoma (NVG) is a potentially blinding secondary glaucoma, characterized by the development of neovascularization of the iris, elevated intraocular pressure (IOP) and, in many instances, poor visual prognosis. In the past, it used was referred to as congestive glaucoma, rubeotic glaucoma or diabetic hemorrhagic glaucoma. In 1963 Weiss and colleagues, proposed the term NVG [1]. Coats first described the histological findings of new vessels on the iris on a patient with central retinal vein occlusion. With the introduction of clinical gonioscopy, the visualization of new vessels in the angle was possible and the origin of elevated IOP was explained by the closure of the iridocorneal angle [1]. There is a high rate of severe visual loss associated with the disease and final visual acuities of hand movements or light perception is not uncommon [1]. Vasconcellos et al. [2] found around 70 % of NVG patients had visual acuity of light perception in a tertiary hospital in Brazil. The incidence of NVG was similar between genders, with slight higher prevalence of men. It more commonly affects the elderly. It was observed that 46.16 % of the patients were between 60 and 79 years of age at onset and 30.68 % were over the age of 80. NVG usually requires not only medication, but also surgical procedures in order to control IOP. The cost of this treatment, both clinical and surgical is often high. In fact, a study in a tertiary hospital in Brazil showed that glaucoma treatment may consume up to 30 % of a family income [3].

### Pathogenesis

NVG is a severe form of glaucoma attributed to new blood vessels obstructing aqueous humor outflow, secondary to posterior segment ischemia [4]. It is associated with the development of a fibrovascular membrane on the anterior surface of the iris and iridocorneal angle of anterior chamber [5]. Invasion of the anterior chamber by a fibrovascular membrane initially obstructs aqueous outflow in an open-angle fashion and later contracts to produce secondary synechial angle-closure glaucoma with high IOP [4]. Iris and angle neo-vessels almost invariably develops before the intraocular pressure rises [5].

Neovascularization is a multi-step process that involves complex interactions of a variety of angiogenic actors. New vessel formation in the eye is affected to a large extent by an unbalance between pro-angiogenic factors (such as, *vascular endothelial growth factor*-VEGF) and other anti-angiogenic factors (such as pigment-epithelium-derived factor) [6].

VEGF plays a major part in mediating active intraocular neovascularization in patients with ischemic retinal diseases [7]. VEGF and insulina growth-1 factors are produced locally in the human eye by a variety of cells including Mueller cells, retinal pigment epithelial cells, retinal capillary pericytes, endothelial cells and ganglion cells [8]. VEGF is sufficient to produce iris neovascularization in a nonhuman primate [9]. Neovascularization was consistent with increased of insulin growth-1 factor and induction of VEGF expression in retinal glial cells. Insulin growth-1 factor accumulated in aqueous humor may cause rubeosis iridis and subsequently adhesions between the cornea and iris may block aqueous humor drainage [10]. Concentration of VEGF can decline after the regression of iris neo-vessels [11]. The non-pigmented ciliary epithelium is an important site of VEGF synthesis in patients with NVG. In fact, a recent study considered the ciliary epithelium as an additional focus of treatment in the management of NVG, especially in eyes that were not responsive to panretinal photocoagulation (PRP) [12].

Others potential pro-angiogenic initiating factors have been investigated in previous studies. The inflammatory cytokine IL-6 concentration in aqueous humor was increased spatially and temporally correlated with the grade of neovascularization of the iris in patients of NVG secondary to central retina vein occlusion [13]. It was also found a possible involvement of basic fibroblast growth factor (bFGF) in the pathogenesis of anterior-segment disorders, such as NVG [14]. Furthermore, increased levels of transforming growth factor-beta 1 and -beta 2 [15], nitric oxide [16] and endothelin-1 [17] in the aqueous humor of patients with NVG was observed. Previous study also suggested a strong correlation with free-radicals such as the superoxide in the aqueous humor of NVG patients [18].

In poorly controlled diabetic patients, with widespread posterior segment ischemia that goes un-recognized and untreated, progression from iris neovascularization to NVG is frequent and can occur after 12-month following the development of iris neovascularization [19] NVD is some diabetic eyes can take a more indolent course, and not immediately result in NVG. In patients with ischemic central retinal vein occlusion, NVG occurs typically between 1.5 to 6 months after ischemic event [20].

### Etiology and diagnosis

The most common causes of NVG are central vein retinal occlusion, proliferative diabetic retinopathy and ocular ischemic syndrome, and central retinal artery obstruction [21].

In Table 1, the conditions that can lead to NVG are summarized and divided by common causes [22–25], uncommon causes, such as those related to ocular tumors [26–30], systemic diseases [31–37] and other rare diseases that can lead to NVG [38–41]. The diagnosis of NVG is clinical and requires detailed patient’s history and a complete ophthalmological examination. Case history is important to determine the origin of ischemia. Patients may be asymptomatic, especially when the IOP rise occurs slowly, or they can present with symptoms such as low vision, ocular pain and photophobia. In the early stages, exam findings can be subtle, requiring the ophthalmologist to maintain high index of suspicion in face of conditions that are commonly associated with NVG such as diabetic retinopathy, central retina vein occlusion or ocular ischemic syndrome [4, 5].

**Table 1:** Summary of conditions that can lead to neovascular glaucoma

Common causes	Ocular tumors
Central retinal vein occlusion	Retinoplastoma
Branch retinal vein occlusion	Uveal melanomas
Proliferative diabetic retinopathy	Ciliary body medulloepithelioma
Caroid arterial obstruction	Vasoproliferative tumors of the retina
Central retinal artery occlusion	Ocular metastasis

Systemic diseases	Other causes
Juvenile myelomonocytic leucemia	Uveitis
Systemic lupus erythematosus	Purtscher’s retinopathy
Juvenile xanthogranuloma	Altered expression of aquaporins
Cryoglobulinemia type 1	Familial amyloid polyneuropathy
Neurofibromatosis type 1	Arteritis from cytomegalovirus retinitis

In diabetic patients, the onset of NVG is generally correlated with poor glycemic control, leading to proliferative diabetic retinopathy and consequently, neovascularization of the anterior segment. Sudden painless visual loss occurring months before, in turn, would be typical related to NVG associated to central retina vein occlusion. NVG may appear between 2 weeks to 2 years after occlusion of the central retinal vein, but most commonly appears after 3 months. History of occlusion of the carotid artery with elevated IOP in ipsilateral eye raises the suspicion of ocular ischemic syndrome. In cases of NVG after CRAO, the onset can be as early as 2 weeks after the onset of artery obstruction [42].

**Fig. 1:**
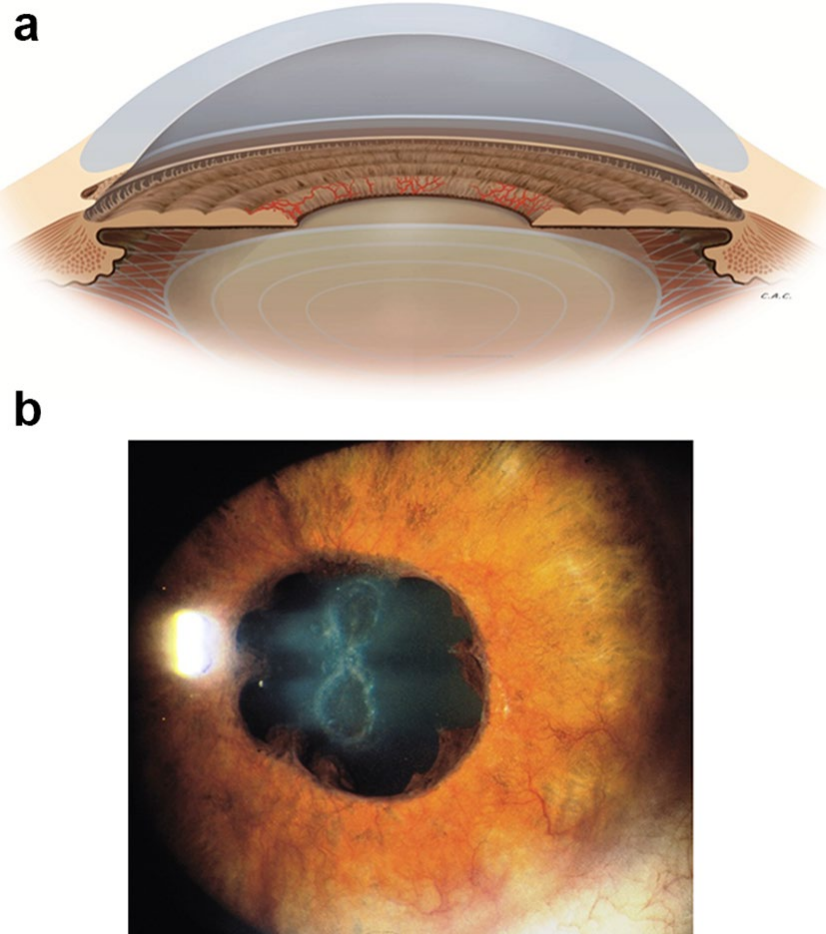
**a** Rubeosis iridis, **b** Anterior chamber in NVG

**Fig. 2:**
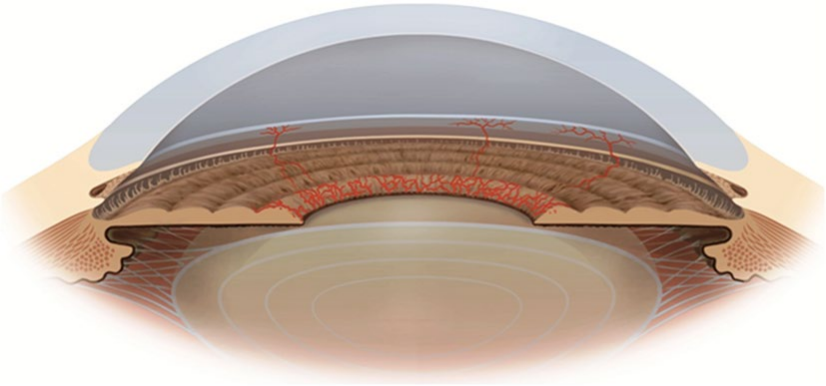
Iris neovessels

On physical examination, a careful examination of iris and anterior chamber angle is essential before pupil dilation for fundus evaluation. Anterior biomicroscopy can reveal: rubeosis iridis (neovessels are vessels that do not follow an organized growth pattern, while iris vessels usually grow radially symmetric), mild anterior chamber reaction, corneal edema due to sharp increase of IOP, ciliary injection and uveal ectropion by contraction of the fibrovascular membrane over the iris (Fig. 1) [4, 5]. Rubeosis starts from the pupillary border with the appearance of tiny tufts of dilated capillaries (Fig. 2) or red spots that can’t be seen unless the iris is examined under high magnification. Rubeosis iridis is usually present, though not always, before neovascularization of the angle. In rare cases, there may be neovascularization of angle without neovascularization of the pupillary border, especially after ischemic central retinal vein occlusion. Therefore, it is important to perform gonioscopy even when the border of the pupil is not involved.

**Fig. 3:**
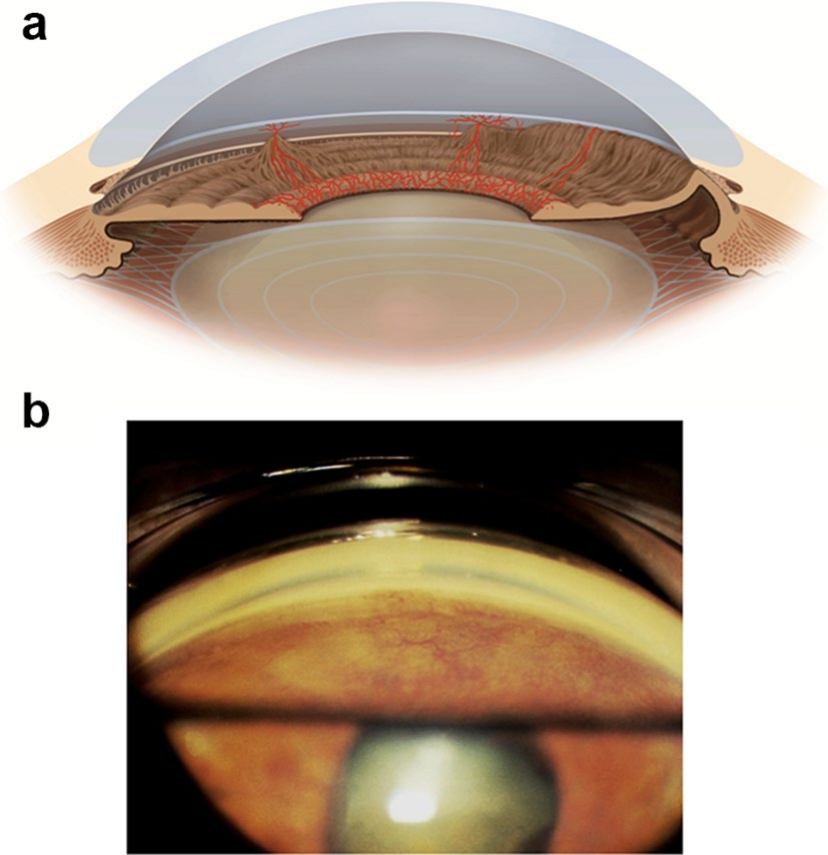
**a** Iridocorneal angle neovessels, **b** Iridocorneal angle neovessels

**Fig. 4:**
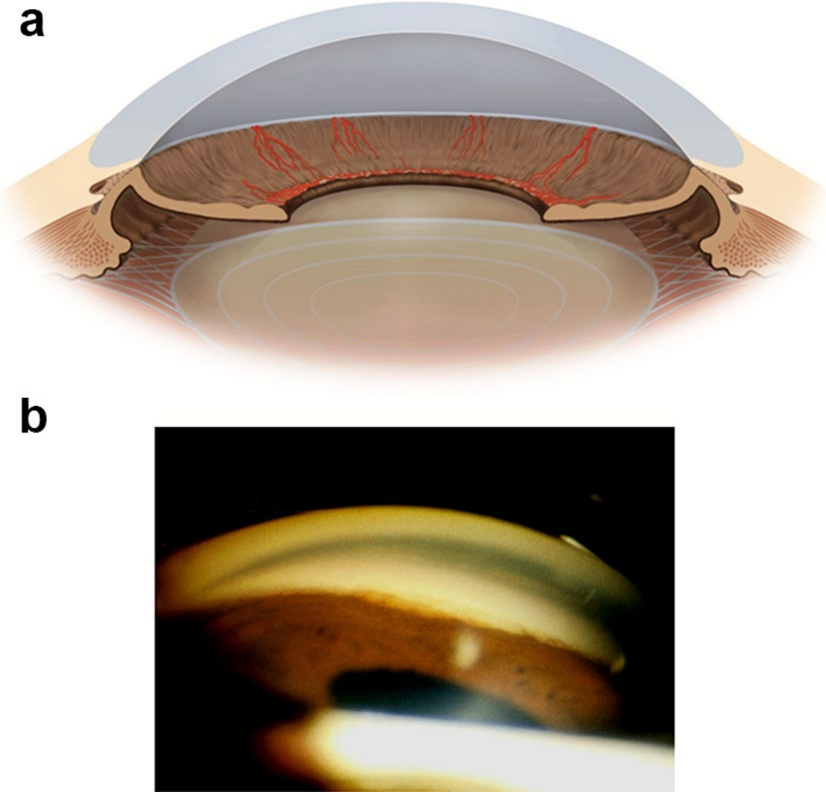
**a** Peripheral anterior synechiae, **b** peripheral anterior synechiae

Initially, the iridocorneal angle appears open on gonioscopy but with the progression of the disease, neovessels can appear over the angle structures (Fig. 3). In the final stages, peripheral anterior synechiae can occur and lead to complete angle closure (Fig. 4) [4, 5]. The IOP may be normal in the early stages of the disease, but usually goes to high levels in advanced stages of the disease when the angle is closed by the contraction of the fibrovascular membrane. On fundus examination, glaucomatous optic nerve damage may already be present depending on the duration of elevated IOP and its levels.

Despite the clinical diagnosis, in some cases, a functional test such as the electroretinography can be used to differentiate between ischemic and non-ischemic forms of central retinal vein occlusion, helping to detect patients more prone to the development of neovascularization of the iris [5]. Both interocular amplitude difference of −23 microV and interocular amplitude ratio of 60 % were good cutoff points to differentiate ischemic from nonischemic central vein retinal occlusion [41] Iris angiography can also be useful in some borderline cases because it shows fluorescein leakage, which is not normally seen [5]. Although these tools can aid in early detection of neovascularization, they are expensive and not always available. In contrast, gonioscopy is a widely available e, fast and low cost procedure that can detect neovascularization of the angle. Retinal angiography may also help diagnosis elucidation, especially in cases of retinal vascular disorders and it can also guide the treatment with retinal photocoagulation. A Doppler ultrasound may be necessary to identify carotid stenosis if obvious retinal ischemia causes are not found [5].

### Clinical treatment

#### Medical treatments

The first step to prevent visual loss and relieve pain or discomfort associated with NVG is to lower the high IOP levels. One of the strategies of medical management of NVG consists of IOP-lowering agents, such as topical β-adrenergic antagonists, α-2 agonists and topical or oral carbonic anhydrase inhibitors. These pharmacologic agents work by suppressing aqueous production and possibly increasing uveoscleral outflow [5, 43]. Prostaglandin analogs should be avoided in order to prevent further breakdown of the blood-aqueous barrier with worsening of the intraocular inflammation [44]. Pilocarpine and other anticholinergic agents are generally contraindicated, because they may increase inflammation, cause miosis, worsen synechial angle closure and decrease uveoscleral outflow. Topical atropine may be used for cycloplegia and might even lower the pressure by increasing the uveoscleral outflow. Atropine also reduces the incidence of hyphema. Since some patients with NVG have some degree of intraocular inflammation, it may helpful to give topical corticosteroids to reduce any inflammatory component that may be present [45]. Oral carbonic anhydrase inhibitors, such as acetazolamide and methazolamide, can be prescribed when topical treatment is not enough to lower IOP [46, 47].

### Photocoagulation

The basis for the treatment of NVG is to reduce posterior segment ischemia and recover the homeostatic balance between pro-angiogenic factors such as VEGF and anti-angiogenic factor, such as the pigment-epithelium-derived factor [6]. Panretinal photocoagulation remains the mainstay in controlling the neovascular drive and should be considered in all cases of NVG when retinal ischemia is present [5]. It is still believed to be helpful in eyes whose angles are already occluded by the new vessels. The procedure is characterized by photocoagulation of the peripheral retina using a slit lamp or indirect laser with 1200–1600 burns and approximately 500 microns spot size. Panretinal photocoagulation is commonl performed over 1–3 sessions. In cases of NVG, the sessions should be peformed as fast as possible. The procedure is usually performed under topical anesthesia. If t topical anesthesia is insufficient, subconjunctival anesthesia or even peribulbar anesthesia can be performed. Panretinal photocoagulation is indicated not only in initial rubeosis, but also in late stages of NVG with goniosynechiae. In cases of cloudy media precluding transpupillary laser, consideration for PRP performed in the operating room during pars plana vitrectomy can be performed. Historically, pan retinal peripheral cryotherapy was done is such cases but this procedure is rarely done now.

This treatment has variable outcome depending on the underlying cause of NVG and also the stage in which the disease was diagnosed. For example, in diabetic retinopathy, after panretinal photocoagulation, complete regression of retinal neovascularization can be reached in 67–77 % of cases, visual loss can be prevented in 59–73 % and IOP reduction can be achieved in 42 % of the cases [48]. If neovascularization persists, additional laser treatment can be performed until complete regression of the neovascularization. In such successfully treated cases of posterior segment neovascularization, anterior segment neovascularization almost never occurs. In central retinal vein occlusion patients, panretinal photocoagulation is indicated in the ischemic form of central retinal vein occlusion due to the high risk of NVG development [49]. Panretinal photocoagulation is also indicated in cases of iris, angle and retinal neovascularization.

The treatment of NVG secondary to ocular ischemic syndrome should be multidisciplinary with the involvement of a cardiologist and/or vascular surgeon for carotid arteries imaging and possible carotid endarterectomy if indicated [24]. Photocoagulation is indicated in ocular ischemic syndrome patients with iris and posterior segment neovascularization to prevent development of secondary NVG. However, is noteworthy to mention that uveal ischemia alone can be responsible for neovascularization and panretinal photocoagulation should be performed if fluorescein fundus angiography shows retinal ischemia due to retinal capillary obliteration [50]. Previous report has suggested that panretinal photocoagulation alone can increase IOP and may compromise optic nerve head circulation. Therefore surgical carotid endarterectomy would be the best treatment in these cases [51].

### Vascular endothelial growth factor inhibitors

Recently, use of anti-VEGF in the management of NVG has been extensively investigated [52]. Since 1996, several studies have been reporting VEGF as an important and predominant factor in the pathogenesis of neovascularization [9, 53]. VEGF inhibitors can stifle the neovascularization process secondary to retinal ischemia [54]. The administration of anti-VEGF is currently becoming established, supported by several studies suggesting better visual prognosis and IOP control following anti-VEGF injections [6, 55].

Anti-VEGF injections can lead to regression of both iris and angle neovascularization, and intraocular pressure control when the angle remains open [56]. However, the effects of anti-VEGF agents seemed to induce only a temporary regression of new vessels in the anterior chamber angle as well as IOP reduction, generally during between four to six weeks [6]. In the current review, we report some of the main results of some studies about use of anti-VEGF in the treatment of NVG.

Yazdani et al. [57] investigated the effect of intravitreal bevacizumab on NVG in a randomized controlled trial with 26 eyes from 26 patients. All eyes received conventional treatment for NVG and were randomly allocated to three 2.5 mg intravitreal bevacizumab injections at 4-week intervals or a sham procedure. Authors concluded that intravitreal injections of bevacizumab reduced iris neovascularization and IOP in NVG and may be considered as an adjunct to more definitive surgical procedures for NVG. In addition, Wittstrom et al. investigated the effect of a single intravitreal injection of bevacizumab for NVG after ischemic central retinal vein occlusion [58]. In this study 19 eyes from 19 patients were randomly allocated to either an intravitreal bevacizumab injection and panretinal photocoagulation (10 eyes) or panretinal photocoagulation alone (9 eyes). Their results suggested that intravitreal injection of bevacizumab might be valuable in the treatment of NVG by improving the resolution of neovascularization.

Liu et al. [59] investigated the efficacy and safety of intravitreal ranibizumab injection combined with trabeculectomy compared it with Ahmed valve surgeries. In this prospective study, they have included 37 eyes from 36 NVG patients, in which 18 NVG eyes were given intravitreal ranibizumab injection one week before trabeculectomy. Ahmed valve implantation surgery was performed in 19 eyes. Their results showed that IOP was significantly decreased following intravitreal ranibizumab injection combined with trabeculectomy treatment. In addition there was a significant, though modest, best-corrected visual acuity improvement in intravitreal ranibizumab injection group. They also had less postoperative complications and lower failure ratio than Ahmed surgery. However, in a recent study conducted by Olmos et al. [60] intravitreal injection of bevacizumab was not superior than panretinal photocoagulation. The study was a retrospective, comparative, case series of 163 eyes of 151 patients with NVG, including 99 treated without and 64 treated with intravitreal bevacizumab. Medical and surgical treatments for NVG were assessed. They found that IOP decreased to 18.3 ± 13.8 mmHg in the non-bevacizumab group and 15.3 ± 8.0 mm Hg in the bevacizumab group. Panretinal photocoagulation substantially reduced the need for glaucoma surgery (*P* < 0.001) in bevacizumab treated NVG eyes. Therefore, although bevacizumab delayed the need for glaucoma surgery, panretinal photocoagulation was the most important factor that reduced the need for surgery. Vision and IOP in eyes with NVG treated with bevacizumab showed no long-term differences when compared with eyes that were not treated with bevacizumab. Thus, intravitreal bevacizumab serves as an effective temporizing treatment, but is not a replacement for close monitoring and definitive treatment of NVG.

A systematic review by Simha et al. [61] found that there is no evidence to evaluate statistically the effectiveness of anti-VEGF treatments, even as an adjunct to conventional treatment in reducing the IOP in NVG. More recently, Tang et al. [62] performed a prospective non-randomized study with 43 eyes of 43 neovascular glaucoma patients. In this study, patients were assigned to receive either 0.5 mg intravitreal ranibizumab for three to 14 days before a Ahmed glaucoma valve implantation (n = 21) or Ahmed glaucoma valve implantation alone (n = 22). They found a success rate of 73.7 vs. 71.4 % at 6 months and 72.2 vs. 68.4 % at 12 months in the injection group and the control group, respectively. There were no significant differences in the two groups with respect to intraocular pressure, best corrected visual acuity, anti-glaucoma medications or postoperative complications at 6 or 12 months. They concluded, therefore that a single intravitreal ranibizumab before surgery has no significant effect on the medium- or long-term outcomes of neovascular glaucoma treated with Ahmed glaucoma valve implantation.

Sahyoun et al. [63] also evaluated the long-term results of the Ahmed glaucoma valve implantation in association with bevacizumab in NVG patients in a retrospective study.

Their study included 39 eyes of 34 patients, which were divided in two groups. The first group consisted of 19 eyes that received an injection of intravitreal bevacizumab 7 days preoperatively, whereas the second group without the injection, included 20 eyes. Even though, preoperative intravitreal bevacizumab before Ahmed glaucoma valve surgery was not associated with a better surgical success, IOP control, or best-corrected visual acuity. Its administration significantly decreased postoperative hyphema and number of last visit’s antiglaucoma medications.

Zhou et al. [64] conducted a systematic review to evaluate the efficacy and tolerability of Ahmed glaucoma valve implantation with intravitreal bevacizumab injection pretreatment in the treatment of NVG.

They found that the intravitreal bevacizumab group was associated with significant greater complete success rates compared with the control group. However, it did not show a significant difference for the qualified success rate between them. In addition, the intravitreal bevacizumab group was associated with a significantly lower frequency of hyphema than the control group.

More recently, newer anti-VEGF agents such as aflibercept have also been used in the treatment of NVG [65]. Soohoo et al. reported a case series study with 4 newly diagnosed stage 1 or 2 NVG patients. They were treated with intravitreal aflibercept at the time of diagnosis, and repeated injections at 4, 8 and then every 8 weeks thereafter up until 52 weeks after study initiation. They found that intravitreal aflibercept resulted in rapid regression of iris and angle neovascularization. IOP was stable or reduced in all patients at the 52-week study visit, suggesting that intravitreal aflibercept may be an effective treatment for stage 1 and 2 NVG, even though further research is needed to determine the full duration of effect and the optimal dose and timing of administration.

In conclusion, there still a debate about the real effectiveness of anti-VEGF in the management of NVG. There is evidence showing that a pre-treatment with anti-VEGF before definitive IOP lowering glaucoma surgeries can significantly lower the frequency of hyphema. But further research is still needed to evaluate the impact on long-term IOP control, visual acuity and cost-effectiveness of the anti-VEGF injections in the management of NVG. It is also important to remember that continuous intravitreal anti-VEGF injections may cause both transient and sustained elevation in IOP [66].

### Surgical treatment

Although the mainstay of therapy of NVG is the treatment of retinal ischemia with panretinal photocoagulation, surgical interventions to control IOP are often necessary since the use of eye-drops may not lower IOP enough to prevent optic nerve damage. Especially in those cases in which peripheral anterior synechia formation and angle closure have occurred. Surgical interventions for NVG include: trabeculectomy with antimetabolites, glaucoma drainage devices, cyclophotocoagulation, among others. NVG is a refractory type of glaucoma that poses a challenge for proper IOP control and is often associated with increased risk for postoperative complications including hyphema and vision loss.

#### Trabeculectomy

NVG has been associated with high rates of failure after trabeculectomy [67, 68] but the adjunct use of antimetabolites has improved the success rate of the surgery [69]. Sisto et al. [69] showed 55 % of success rate in a mean follow-up of 35 months with the use of postoperative 5-fluorouracil and 54 % of success rate in a mean follow-up 18 months with intraoperative mitomycin C. Still, compared to other types of glaucoma, NVG is a known risk factor for surgical failure [70]. Moreover, it has been suggested that a postoperative hyphema, a common complication in patients with NVG, may be associated with higher rates of trabeculectomy failure in NVG [71].

#### Glaucoma drainage devices

Glaucoma drainage devices are usually considered the first treatment option for refractory glaucoma. However, NVG patients are at greater risk for surgical failure after Ahmed glaucoma valve surgery compared with controls. Yalvac reported 63.2 and 56.2 % of success rates at 1 and 2 years after Ahmed glaucoma valve implantation, respectively [72]. Hernandez-Oteyza recently reported a success rate of 60 % at 1 year of follow-up and found that a hypertensive phase in the postoperative period and a worse preoperative BCVA to be risk factors for Ahmed valve surgical failure in patients with NVG [73]. Netland et al. found that the success rate was significantly lower over time in eyes with NVG compared with controls. They reported success rates at 5 years of 81.8 % for control and 20.6 % for patients with NVG [74]. Similar results have been reported with other types of glaucoma drainage devices [75–78]. Furthermore, there is no evidence of improved surgical outcomes with glaucoma drainage devices as opposed to augmented trabeculectomy. Similar results have been reported when treatment with Ahmed Glaucoma valve was compared to trabeculectomy with mitomycin C. Shen et al. reported success rates of 70 and 65 % at 1 year and 60 and 55 % at 2 years after Ahmed glaucoma valve and trabeculectomy with mitomycin C, respectively [79]. Therefore, proper control of retinal neovascularization in addition to either trabeculectomy with mitomycin C or glaucoma drainage device implantation seem appropriate treatment options for IOP control in NVG patients.

A randomized clinical trial by Arcieri et al. investigated the efficacy and safety of intravitreal bevacizumab in eyes with NVG undergoing Ahmed glaucoma valve implantation. They enrolled 40 patients who were randomized to receive intravitreal bevacizumab (1.25 mg) or not during Ahmed valve implant surgery. Injections were administered intra-operatively, 4 and 8 weeks after surgery. Their results suggest a trend that using with intravitreal bevacizumab as an adjunct can lower IOP levels and the number of post operative medications in NVG patients who underwent Ahmed glaucoma valve implantation. It is important to note, however, that patients with NVG are at a higher risk for certain postoperative complications and poor visual outcomes, possibly due to progression of underlying disease. Loss of light perception is not rare among NVG patients after surgical procedures [74, 75, 77] and hyphema is often encountered [80]. Compared to other types of glaucoma, NVG eyes also seem to be at higher risk for tube shunt exposure [81].

Since NVG and proliferative diabetic retinopathy are usually co-existing conditions, it is not uncommon for patients with NVG to have a positive history of prior vitrectomy. Studies that evaluated implantation of Ahmed glaucoma valve for IOP control in vitrectomized eyes, showed the safety and efficacy of the procedure [82, 83], with success rates of 62.5 % after 3 years for vitrectomized eyes, which was not statistically different from the 68.5 % success rate for the nonvitrectomized group. Ahmed glaucoma valve can control the IOP in the majority of eyes after pars plana vitrectomy and silicone oil injection, when implanted in the anterior chamber or inferonasal or inferotemporal quadrant, preventing oil to clogging the tube. [84]. If this surgery is selected, intra-silicone injection of anti-VEGF in posterior segment for regressing iris neovascularization is considered safe and effective [85]. However, intraocular silicone oil tamponade was found to be a risk factor for surgical failure [83]. The combination of 23-gauge pars plana vitrectomy and Ahmed valve implantation in the same procedure is also a treatment option for these cases and has been shown to be safe and effective in patients with proliferative diabetic retinopathy and refractory NVG [86, 87]. Wallsh et al. confirmed these findings in a retrospective study with a 22 patients, in which 95.8 % of eyes had IOP below 21 mmHg in the final follow-up (mean follow-up of 7.39 ± 1.11 months). Best-corrected visual acuity also improved significantly [88]. Finally, a retrospective study evaluated the results of combined pars plana vitrectomy and pars plana Baerveldt tube placement. A significant IOP decrease was achieved with the procedure while visual acuity remained unchanged. However, it is important to note that 38 % experienced a decrease in vision [89] However, prospective and comparative studies with longer follow-up are still needed.

### Cyclodestructive procedures

Transcleral application of diode laser cyclophotocoagulation consists of the destruction the ciliary body epithelium and stroma with consequent reduction of aqueous humor production and IOP levels [90–92]. Transcleral cyclophotocoagulation with and without the use anti-VEGF has been shown to be effective in lowering IOP and relieving pain in advanced cases of NVG [70, 93–95]. When compared to Ahmed valve implantation in a randomized controlled trial, no significant difference was found in the success rate at 24 months between the diode cyclophotocoagulation (61.18 %) and Ahmed glaucoma valve implantation (59.26 %) in NVG treatment [91]. It is important to note, however, that the underlying diagnosis of NVG poses an increased risk for hypotony after transcleral-cyclophotocoagulation [94–97]. Endo-cyclophotocoagulation was also shown to be effective in NVG. A study showed success rates at 24 months of 70.59 and 73.53 % for the Ahmed and endo-cyclophotocoagulation groups, respectively [98].

### Other surgical options

Due to the relatively low long-term success rates of the existing treatment options for NVG, new surgical approaches have been proposed for IOP control. For example, manual and bimanual maneuvers to remove the fibrovascular membrane from the anterior chamber angle have been described [99]. The use of drainage devices made of porous material such as the Ahmed M4 [100]—and the Express shunt [101] has also been attempted. However, more studies and randomized clinical trials are needed to assess the efficacy of such procedures.

## Conclusion

NVG is an important secondary glaucoma associated with poor visual prognosis, due to the optic nerve damage from high IOP and also complications from retinal vascular diseases. Even though treatment options with panretinal photocoagulation and anti-VEGF might be used in attempt to control the neovascularization process, in some cases surgical procedures are necessary in order to achieve normal levels of IOP and avoid optic nerve damage. Proper management and early diagnosis of this condition is crucial to reduce the chances of visual impairment.

## Abbreviations

NVG: Neovascular glaucoma
IOP: Intraocular pressure
VEGF: Vascular endothelial growth *factor*
